# 11% Organic Photovoltaic Devices Based on PTB7‐Th: PC_71_BM Photoactive Layers and Irradiation‐Assisted ZnO Electron Transport Layers

**DOI:** 10.1002/advs.201700858

**Published:** 2018-05-21

**Authors:** Havid Aqoma, Sujung Park, Hye‐Yun Park, Wisnu Tantyo Hadmojo, Seung‐Hwan Oh, Sungho Nho, Do Hui Kim, Jeonghoon Seo, Sungmin Park, Du Yeol Ryu, Shinuk Cho, Sung‐Yeon Jang

**Affiliations:** ^1^ Department of Chemistry Kookmin University Seoul 02707 Republic of Korea; ^2^ Department of Physics and EHSRC University of Ulsan Ulsan 44610 Republic of Korea; ^3^ Department of Chemical and Biomolecular Engineering Yonsei University Seoul 03722 Korea; ^4^ Radiation Research Division for Industry and Environment Korea Atomic Energy Research Institute (KAERI) Jeollabuk‐do 56212 Republic of Korea

**Keywords:** charge extraction, electron transporting layers, irradiation, organic photovoltaics, zinc oxide

## Abstract

The enhancement of interfacial charge collection efficiency using buffer layers is a cost‐effective way to improve the performance of organic photovoltaic devices (OPVs) because they are often universally applicable regardless of the active materials. However, the availability of high‐performance buffer materials, which are solution‐processable at low temperature, are limited and they often require burdensome additional surface modifications. Herein, high‐performance ZnO based electron transporting layers (ETLs) for OPVs are developed with a novel *g*‐ray‐assisted solution process. Through careful formulation of the ZnO precursor and *g*‐ray irradiation, the pre‐formation of ZnO nanoparticles occurs in the precursor solutions, which enables the preparation of high quality ZnO films. The *g*‐ray assisted ZnO (ZnO‐G) films possess a remarkably low defect density compared to the conventionally prepared ZnO films. The low‐defect ZnO‐G films can improve charge extraction efficiency of ETL without any additional treatment. The power conversion efficiency (PCE) of the device using the ZnO‐G ETLs is 11.09% with an open‐circuit voltage (*V*_OC_), short‐circuit current density ( *J*_SC_), and fill factor (FF) of 0.80 V, 19.54 mA cm^‐2^, and 0.71, respectively, which is one of the best values among widely studied poly[4,8‐bis(5‐(2‐ethylhexyl)thiophen‐2‐yl)benzo[1,2‐b;4,5‐b′]dithiophene‐2,6‐diyl‐alt‐(4‐(2‐ethylhexyl)‐3‐fluorothieno[3,4‐b]thiophene‐)‐2‐carboxylate‐2‐6‐diyl)]: [6,6]‐phenyl‐C_71_‐butyric acid methyl ester (PTB7‐Th:PC_71_BM)‐based devices.

## 1 Introduction

Organic photovoltaics (OPVs) have attracted considerable attention due to their potential as low‐cost flexible power generators.^1^, ^2^, ^3^ During the last few decades, efforts to improve the power conversion efficiency (PCE) have been the major focus of the field of OPVs by synthesizing new photoactive materials,^4^, ^5^, ^6^ optimizing the nanoscale morphology in active layers,^7^, ^8^, ^9^ and engineering the interfacial charge extraction properties.^10^, ^11^, ^12^ Enhancement of the interfacial charge collection efficiency has been a cost‐effective way to improve the performance of OPVs because it is universally applicable regardless of the active materials.^13^, ^14^, ^15^

Charge transporting/selecting layers (or buffer layers) have been widely used to improve the interfacial charge extraction properties. They often increase the internal electric field, which is otherwise limited by the Fermi‐level pinning between the active layers and the electrodes. Charge extraction has often been improved by reducing the interfacial charge recombination. Solution‐processed metal oxides (MOs) are widely used buffer materials, which can efficiently modify the active/electrode interfaces. However, their performance is not always optimal;^14^, ^16^ thus, additional surface treatments of the MO layers have often been used to further optimize the interfacial properties. The construction of electric dipole layers using a polyelectrolyte,^17^, ^18^, ^19^ polar solvent,^11^, ^20^ alkali‐metal carbonate,^10^, ^21^ or self‐assembled monolayer^22^, ^23^ has effectively tuned the work functions (WFs) by shifting the vacuum energy level (*E*_vac_) at the interfaces enabling an improved charge transfer. While those aforementioned methods provide positive effects on device performance, their availability has often been limited due to the difficulty in securing the suggested materials. Even when using commonly available materials, it becomes burdensome because an extra coating and/or annealing steps are required.

In addition to the interfacial issues at MO/active layer that has been frequently highlighted, the intrinsic quality of MOs is strongly governed the charge transporting properties.^24^, ^25^ Particularly, in MOs prepared by wet‐chemical solution processes (typically sol–gel conversions), the prevailing defects sites act as charge recombination centers reducing the charge collection efficiency.^26^, ^27^ Recently, there have been a growing consensus to improve the charge transporting properties of MOs by the passivation of the defect sites. In our earlier report, the charge collection in OPVs was improved through the surface defect passivation of solution‐prepared ZnO layers using an ultrathin ZnO layer by the atomic layer deposition (ALD) method.^28^ Chemical treatment^29^ and modification using oxygen containing polymers^30^, ^31^ has achieved effective passivation of the surface defects of ZnO resulting in an enhanced charge collection. The doping of ZnO using heteroatoms effectively enhanced charge collection efficiency by improving carrier concentration and matching favorable energy levels. However, it often required vacuum process (i.e., atomic layer deposition and chemical vapor deposition), and/or high‐temperature annealing (300–400 °C).^32^, ^33^, ^34^, ^35^ Moreover, the reduction of surface defects was not always effectively achieved. The development of techniques to improve the intrinsic quality (i.e., low defects sites) of low‐temperature solution‐processed MOs without additional surface treatment processes will be a universal benefit for device performance and processing.

Herein, we developed low‐defect‐density ZnO‐based electron transporting layers (ETLs) using a solution‐process assisted by a radio‐chemical method. It is known that radiation‐induced processes (also called radiation chemistry or radiochemistry), which use ionized radiation such as an electron beam, γ‐ray, or X‐ray, have successfully been used in the modification of conventional chemical methods for the preparation of materials. These processes are mostly temperature independent and have yielded materials with high purity and uniformity.^36^, ^37^ In this work, high‐quality (i.e., low‐defect‐density) ZnO films were prepared by in situ sol–gel conversion of γ‐ray irradiated ZnO precursor solutions at a low‐temperature annealing (130 °C). The application of the γ‐ray‐assisted‐ZnO (ZnO‐G) layers as ETLs for OPVs showed a remarkably improved interfacial charge extraction compared to conventionally prepared ZnO. The OPV devices using ZnO‐G as the ETL and poly[4,8‐bis(5‐(2‐ethylhexyl)thiophen‐2‐yl)benzo[1,2‐b;4,5‐b′]dithiophene‐2,6‐diyl‐alt‐(4‐(2‐ethylhexyl)‐3‐fluorothieno[3,4‐b]thiophene‐)‐2‐carboxylate‐2‐6‐diyl)] (PTB7‐Th): [6,6]‐phenyl‐C_71_‐butyric acid methyl ester (PC_71_BM) bulk‐heterojunction as the active layers achieved a remarkably high PCE of 11.09%, whereas the devices using the conventional ZnO ETL had a PCE of 8.41%. This result is one of the highest PCEs among the widely studied PTB7‐Th:PC_71_BM devices.

## 2 Results and Discussion

**Figure 1:**
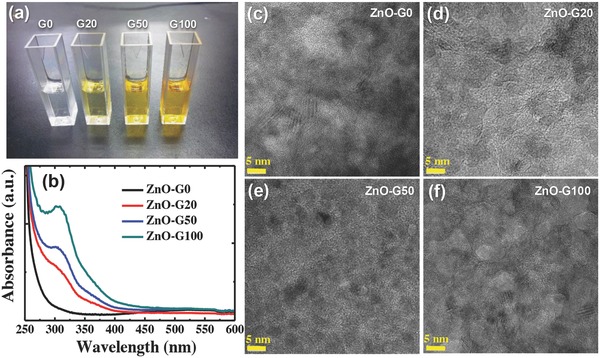
The effects of γ‐ray irradiation on the ZnO precursor solutions; a) photoimages of the precursor solutions, b) UV–vis spectra of the precursor solutions, and c–f) TEM images of various ZnO films.

**Figure** **1**a shows the photoimages of the ZnO precursor solutions by γ‐ray irradiation. The pristine precursor solutions comprised of 0.45 m zinc acetate dehydrate (Zn(CH_3_COO)_2_·2H_2_O) in 2‐methoxyethanol with ethanolamine (NH_2_CH_2_CH_2_OH) was transparent and colorless. By γ‐ray irradiation from 20 to 100 kGy, the color of the precursor solution gradually became yellowish, which was also confirmed by the UV–vis absorption spectra in Figure 1b. For convenience, we denote the irradiated precursor solutions as G0, G20, G50, and G100, in which the numbers indicate the applied irradiation energy in units of KGy. The irradiated precursor solutions (G20, G50, and G100) exhibited a shoulder at ≈310 nm, whereas the pristine precursor solution (G0) had no absorption feature >280 nm. The intensities of the shoulders near 310 nm were gradually enhanced as the radiation energy increased. Notably, the G100 solution showed a very similar absorption spectrum to the crystalline ZnO nanoparticles.^38^, ^39^, ^40^ This result suggests that the γ‐ray irradiation induced the growth of ZnO nanoparticles in the precursor solutions.

It is known that MO nanoparticles can be formed by ionizing energy inducing reduction processes.^36^ Upon irradiation of ionizing energy, reductive hydrated electrons (e_aq_^−^), for which the reduction potential is known to be *E*_0_ = −2.87 V, are generated (1).^36^ On the other hand, a hydroxyl radical (·OH), an oxidation agent with an oxidative potential of +2.73 V, is also generated and competes with the reduction by hydrated electrons. In order to prevent the oxidation, we used zinc acetate in 2‐methoxyethanol for the formulation of the precursor solution because formate ions (HCOO^−^) can scavenge the ·OH forming strong reducing agent, carbon dioxide ion radical (·COO^−^, *E*_0_ = −1.90 V) (2), while the 2‐methoxyethanol reacts with ·OH to form a hydroxyalkyl radical (*E*_0_ = −1.39 V) (3). As a result, the reduction of the ZnO precursor to ZnO nanoparticles is promoted (4) and (5). Using this concept, we successfully prepared the preformed ZnO nanoparticles in the precursor solutions.

(1) $$H_{2}O\;\overset{hv}{\to }\;e_{\mathrm{aq}}^{-},\;H_{3}O^{+},\;H\cdot ,\;O\cdot H$$

(2) $${\mathrm{HCOO}}^{-}\;+\;O\cdot \mathrm{H/H}\cdot \;\to \;C\cdot {\mathrm{OO}}^{-}\;+\;H_{2}{\mathrm{O/H}}_{2}$$

(3) $${\mathrm{CH}}_{3}{\mathrm{OCH}}_{2}{\mathrm{CH}}_{2}\mathrm{OH}\;+\;O\cdot \mathrm{H/H}\cdot \;\to \;{\mathrm{CH}}_{3}{\mathrm{OCH}}_{2}C\cdot \mathrm{HOH}\;+\;H_{2}{\mathrm{O/H}}_{2}$$

(4) $${\mathrm{Zn}}^{2+}\;+\;2e_{\mathrm{aq}}^{-}/2C\cdot {\mathrm{OO}}^{-}/2{\mathrm{CH}}_{3}{\mathrm{OCH}}_{2}C\cdot \mathrm{HOH}\;\to \;\mathrm{Zn}$$

(5) $$2\mathrm{Zn}\;+\;O_{2}\;\to \;2\mathrm{ZnO}$$

**Figure 2:**
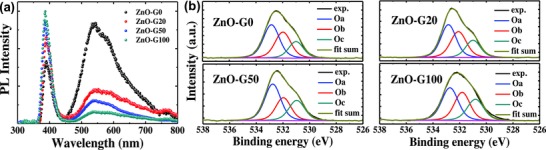
a) PL spectra and b) O 1*s* core level XPS spectra of various ZnO films.

To prepare ZnO films using the γ‐ray irradiated precursor solutions, we spin coated the precursor solutions and annealed them at a low‐temperature (130 °C for 20 min) for additional sol–gel conversion. For convenience, we denote the resulting ZnO films from the various irradiated precursor solutions as ZnO‐G0, ZnO‐G20, ZnO‐G50, and ZnO‐G100. The high‐resolution transmission electron microscopy (TEM) images in Figure 1c–f show the nanoscale crystals in the ZnO films. The density of the nanocrystals was highest in the ZnO‐G100 films among the studied ZnO films. This result revealed that the preformed ZnO nanocrystals in the precursor solutions by γ‐ray irradiation facilitated the ZnO nanocrystal formation during the low‐temperature sol–gel conversion. To elucidate the quality of the resulting ZnO films, we first measured the photoluminescence (PL) spectra (**Figure** **2**a) because the defect‐related PL emission at ≈550 nm is a critical tool for direct determination of the defect density of ZnO.^41^, ^42^, ^43^, ^44^, ^45^, ^46^ As shown in Figure 2a, the ZnO‐G100 shows a substantially lower defect‐related green emission than that of the ZnO‐G0. This result revealed that the ZnO‐G100 contains the smallest grain boundary within the densely packed nanocrystals, and the other irradiation‐assisted ZnO films (ZnO‐G50 and ZnO‐G20) showed a lower defect PL emission than that of the untreated pristine ZnO (ZnO‐G0).

To further investigate the surface properties of the ZnO films, X‐ray photoelectron spectroscopy (XPS) analysis was carried out. The wide‐range XPS spectra are shown in Figure S1 in the Supporting Information. Generally, the major causes of defects in solution‐prepared ZnO are nonlatticed oxygen containing species on the surface. Figure 2b shows the O 1*s* core level spectra, which are deconvoluted into three peaks using the Gaussian function.^47^, ^48^ The highest‐binding‐energy peak at 532.8 ± 0.1 eV (*O*_a_) is generally assigned to nonstoichiometric near‐surface oxygen, surface hydroxylation, adsorbed H_2_O, or adsorbed O_2_ from air. The middle‐binding‐energy peak at 532.1 ± 0.1 eV (*O*_b_) is attributed to lost oxygen ions in deficient regions (vacancies) within the ZnO matrix. The lowest‐binding‐energy‐peak at 531 ± 0.1 eV (*O*_c_) corresponds to stoichiometric oxygen atoms in the ZnO structure (i.e., O–Zn bonding). The relative ratio of each component derived from this XPS analysis is summarized in Table S1 in the Supporting Information. The defect ratio can be inferred by the following relation: ρ_D_ = *O*_a_ + *O*_b_/*O*_c_. The calculated ρ_D_ for the ZnO‐G0 was 3.84 while it decreased to 2.79 for the ZnO‐G100. This result combined with the TEM and PL analysis results indicate that the crystallinity of the ZnO was enhanced while the defect sites were reduced in the ZnO‐G films. To further investigate the crystallinity of ZnO films, grazing incidence X‐ray diffraction (GIXRD) measurement was performed. Figure S2 in the Supporting Information shows the sufficiently developed (002) and (100) ZnO phases were detected in the low‐temperature annealed ZnO films.^49^

The Fermi energy levels of ZnO films were measured by ultraviolet photoelectron spectroscopy (UPS). As shown in Figure S3 in the Supporting Information, there was no significant alteration in work function of ZnO due to irradiation. To investigate the effect of defect reduction in the electric properties of ZnO films, we determined the electron mobility (µ_e_) by measuring space‐charge limited current (SCLC) of electron‐only devices (indium‐doped tin oxide (ITO)/ZnO/LiF/Al) (Figure S4, Supporting Information). The γ‐ray irradiated ZnO films show higher µ_e_ compared to pristine ZnO. The µ_e_ values of ZnO films by SCLC were 5.62 × 10^−4^, 1.12 × 10^−3^, 1.35 × 10^−3^, and 1.56 × 10^−3^ cm^2^ v^−1^ s^−1^ for ZnO‐G0, ZnO‐G20, ZnO‐G50, and ZnO‐G100, respectively. As shown in scanning electron microscopy (SEM) images (Figure S5, Supporting Information) and atomic force microscopy (AFM) images (Figure S6, Supporting Information), the surface morphology of ZnO films with and without irradiation was similar, which indicates the surface morphology is not the cause of performance alteration.

**Figure 3:**
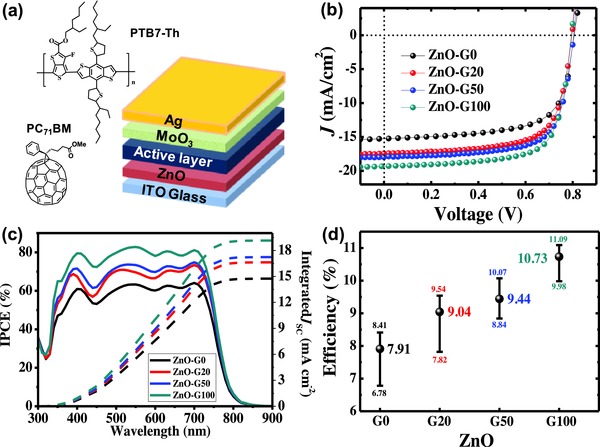
Photovoltaic performance of the OPV devices using ZnO‐G ETLs; a) Schematic illustrations of an inverted OPV device and the active materials used in this study, b) *J–V* characteristics of the devices, c) IPCE spectra of the devices, and d) statistical results of the performances of the devices.

Motivated by the high quality of the ZnO‐G films, we fabricated inverted‐structure OPV devices (ITO/ZnO‐G/PTB7‐Th:PC_71_BM/MoOx/Ag) using the various ZnO films as ETLs. Bulk‐heterojunctions of PTB7‐Th and PC_71_BM were used as the active materials. **Figure** **3**a shows a schematic illustration of the device architecture as well as the chemical structures of the photoactive materials. Figure 3b exhibits the current density‐voltage (*J*–*V*) characteristics of the inverted OPV devices. The optimized devices using the pristine ZnO layer (ZnO‐G0) showed a PCE of 8.41% with an open circuit voltage (*V*_OC_) of 0.81 V, a short circuit current (*J*_SC_) of 15.27 mA cm^−2^, and a fill factor (FF) of 0.68, which is comparable to other reported results.^50^, ^51^, ^52^, ^53^ The device performance was improved by using the ZnO‐G‐based ETLs. The PCE of the ZnO‐G20‐based devices improved to 9.54%, while that of the ZnO‐G50‐based devices was 10.07%. The device performance was optimized when the ZnO‐G100 was used; the PCE was 11.09% with a *V*_OC,_ *J*_SC_, and FF of 0.80 V, 19.54 mA cm^−2^ and 0.71, respectively. The detailed parameters of the *J–V* characterization results are summarized in **Table** **1**. The consistency of the device performance was confirmed by statistical analysis (20 devices for each condition) shown in Figure 3d. The incident‐photon‐to‐current‐efficiency (IPCE) spectra in Figure 3c revealed that the significant external quantum efficiency (EQE) improvement in the entire visible range is responsible for the higher PCE in the ZnO‐G‐based devices. Because identical active layers were used for all the studied devices, and no significant optical effect by various ZnO‐G films (Figure S7, Supporting Information), the change in the EQE must come from the different charge collection efficiencies. The mismatch of the *J*_SC_ values obtained from the *J–V* characteristics and estimated from EQE spectra was <4% in all devices confirming the validity of the PCE and *J*_SC_ values. Notably, the PCE of our devices using the ZnO‐G100 is one of the highest values recorded among the widely studied PTB7‐Th:PC_71_BM‐based devices (Table S2, Supporting Information).

**Table 1:** Summary of the performances of the OPV devices

ZnO ETL	PCE [%]	*V*_OC_ [V]	*J*_SC_ [mA cm^−2^]	FF	Calculated *J*_SC_ [mA cm^−2^]
ZnO‐G0	8.41	0.81	15.27	0.68	14.74
ZnO‐G20	9.54	0.80	17.29	0.69	16.63
ZnO‐G50	10.07	0.80	17.98	0.70	17.23
ZnO‐G100	11.09	0.80	19.54	0.71	19.14

**Figure 4:**
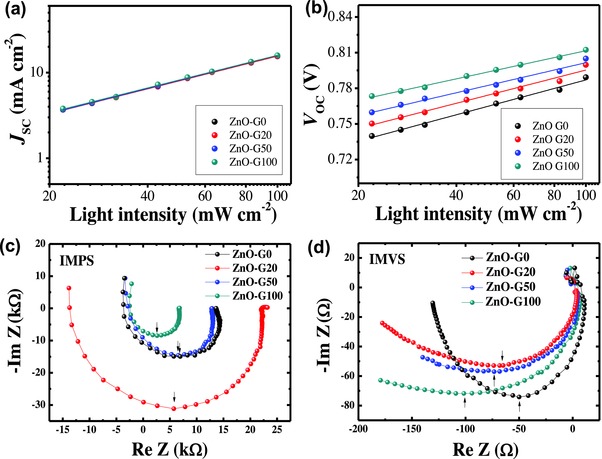
a) *J*_SC_ as a function of the light intensity and the power‐law fit and b) *V*_OC_ as a function of the light intensity and the logarithmic fit. Charge collection efficiency analysis of the devices by c) IMPS and d) IMVS.

To further understand the improved EQE of the ZnO‐G‐based devices, the charge extraction properties were investigated. **Figure** **4**c,d shows the analysis results by intensity modulated photocurrent spectroscopy (IMPS) and intensity modulated photovoltage spectroscopy (IMVS), respectively. The detailed parameters obtained from the IMPS/IMVS are summarized in **Table** **2**. The charge transport time (τ_ct_) was estimated from the results of the IMPS analysis using the following relation: τ_ct_ = 1/2π *f*_min(IMPS)_, where *f*_min_ is the minimum current of the imaginary part of the low‐frequency range in the IMPS spectra (Figure 4c). The charge recombination lifetime (τ_r_) was estimated from the results of the IMVS analysis (Figure 4d) using the following relation: τ_r_ = 1/2π *f*_min(IMVS)_, where *f*_min_ is the minimum voltage of the imaginary part of the low‐frequency range of the IMVS spectra. The τ_ct_ value of the ZnO‐G0 devices (0.45 µs) was improved to 0.57 µs in the ZnO‐G100 devices indicating a facilitated charge transport. More significantly, the τ_r_ value of the ZnO‐G100‐based devices was sixfold higher (18.8 µs) compared to the ZnO‐G0‐based devices (3.18 µs). As a result, the estimated charge collection efficiency (η_c_), which is obtained by the relation η_c_ = 1/(τ_ct_/τ_r_),^54^, ^55^ of the ZnO‐G0 devices (0.821) was substantially improved to 0.976 in the ZnO‐G100 devices. This result confirmed that the charge carrier loss in the devices using γ‐ray assisted ZnO‐ETLs was considerably suppressed because of the reduced defect sites.^28^, ^43^, ^56^, ^57^

**Table 2:** Summary of the characterization results of the OPV devices

ZnO ETL	Light intensity measurement		IMVS		IMPS		Charge collection efficiency
	α	*kT*/*q*	*f*_min_ [Hz]	*τ_r_* [µs]	*f*_min_ [Hz]	τ_t_ [µs]	η_c_
ZnO‐G0	0.96	1.25	5.01 × 10^4^	3.18	2.82 × 10^5^	0.57	82.1
ZnO‐G20	0.96	1.20	1.75 × 10^4^	9.1	2.86 × 10^5^	0.56	93.8
ZnO‐G50	0.97	1.08	1.25 × 10^4^	12.7	3.16 × 10^5^	0.5	96.1
ZnO‐G100	0.96	1.01	8.47 × 10^3^	18.8	3.55 × 10^5^	0.45	97.6

In order to gain more insight into the charge recombination behaviors in the OPV devices, we measured the changes in the *J*_SC_ and *V*_OC_ under various illumination intensities (Figure 4a,b). The power law dependence of *J*_SC_ upon the light intensity can be expressed as *J*_SC_ ∝ *I^α^*, where *I* is the illumination intensity and α is the exponential factor.^58^, ^59^ Given that the α value is close to unity, bimolecular recombination during the charge sweep‐put under short‐circuit conditions is negligible.^59^, ^60^, ^61^ The α values of all the studied devices were similarly high (0.96–0.97), indicating no significant bimolecular recombination at the short‐circuit condition (Figure 4a). However, the slope of *V*_OC_ changes as a function of the illumination intensity showed a considerable change (Figure 4b). The slope of *V*_OC_ versus light intensity gives *k*T/*q*, where *k*, T, and *q* are the Boltzmann constant, temperature in Kelvin, and the elementary charge, respectively.^61^, ^62^ The larger the *k*T/*q* value, the greater the probability of trap‐assisted recombination.^63^ The slope of the plot for the ZnO‐G100 devices was lower (1.01*k*T/*q*) than that of the ZnO‐G0‐based devices (1.25*k*T/*q*). This result confirmed that the reduced defect density in ZnO‐G100 compared to that in ZnO‐G0 suppressed the interfacial trap‐assisted recombination at the open‐circuit condition. The *J*_SC_ and *V*_OC_ versus the illumination intensity characterization revealed that the reduced defect density in the γ‐ray assisted ZnO layers diminished the interfacial trap‐assisted charge recombination in the devices.

**Figure 5:**
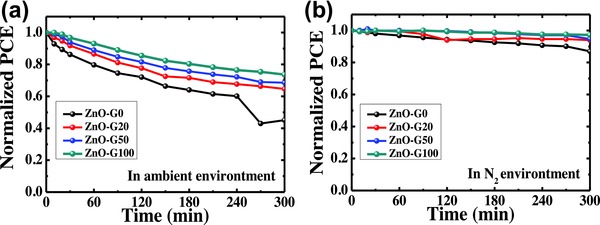
Stability under continuous illumination of various devices in a) ambient and b) N_2_ environment.

The photostability of devices under continuous illumination in ambient and nitrogen environment was monitored (**Figure** **5**). In ambient condition, the ZnO‐G100 device retain >70% of its initial PCE, which is significantly higher than pristine ZnO device (retain ≈40% of PCE). In nitrogen condition, the performance degradation of devices was reduced due to decreased photo‐induced oxidation. The ZnO‐G100 device retain >90% of its initial PCE after 5 h of illuminations, whereas the pristine ZnO device retain only ≈80%. This result indicates that the reduced surface defects in irradiated ZnO improved the photostability of devices.

## 3 Conclusion

We developed high‐performance ZnO‐based ETL materials using a radio‐chemically assisted solution‐process. The proper formulation of the precursor solution and γ‐ray irradiation preformed the ZnO nanoparticles which aided in the fabrication of high‐quality ZnO‐G films by low‐temperature sol–gel conversion. The resulting ZnO‐G films contained a substantially low defect density compared to conventional solution‐processed ZnO films, which can efficiently reduce the interfacial trap‐assisted recombination. The charge collection efficiency of the OPVs was remarkably improved, and the devices using the ZnO‐G as an ETL exhibited approximately a 32% higher PCE than that of the devices using the conventional solution‐processed ZnO ETLs. The PCE of 11.09% from our OPV devices was one of the best values among the widely studied PTB7‐Th:PC_71_BM‐based devices.

## 4 Experimental Section

*Preparation of the ZnO‐G Films*: The ZnO sol–gel precursor solution was prepared by dissolving zinc acetate dehydrate (Zn(CH_3_COO)_2_.2H_2_O) and ethanolamine in 2‐methoxyethanol (0.45 m), followed by vigorous stirring at 80 °C for 6 h. The ZnO sol–gel precursor (30 mL) was added to the vial (30 mm of diameter and 110 mm of height) prior to γ‐ray irradiation. The γ‐ray radiation facility is equipped with ^60^Co source (ACEL type C‐1882, MDS Nordion, Canada) and performed in Korea Atomic Energy Research Institute with perfect radiation shielding. The ZnO precursor solutions were treated by γ‐ray irradiation with different radiation intensities at a dose rate of 10 kGy h^−1^. Note that sampling must be handled with care and appropriate safety equipment due to exposure of high‐energy radiation. For the preparation of the ZnO‐G films, the ZnO precursor solutions were spin coated for 15 s at 4000 rpm onto ITO/glass substrates followed by thermal annealing at 130 °C for 20 min.

*Device Fabrication*: The organic active layer solution was prepared by dissolving PTB7‐Th (8 mg, 1‐Material) and PC_71_BM (12 mg, EM‐Index) in chlorobenzene (0.97 mL) and 1,8‐diiodooctane (0.03 mL). The solution was stirred at 70 °C for 2 h and then filtered through a polytetrafluoroethylene filter (2 µm pore size, Whatman). The deposition of the active layers was performed onto ZnO/ITO/glass substrate by spin coating at 1000 rpm for 60 s, and the resulting thickness of the active films was ≈110 nm. Finally, MoOx (8 nm) and Ag (100 nm) were deposited under a high vacuum (<10^–6^ Torr). The nominal device active area defined by the overlap of the anode and cathode was 0.0707 cm^−2^.

*Device Characterizations*: Measurements of the *J*–*V* characteristics were performed using a Keithley 2401 source unit and simulated solar radiation of AM 1.5G one sun illumination under ambient air condition. The light intensity was adjusted using a monosilicon detector with a KG‐5 filter calibrated at the National Renewable Energy Laboratory to minimize spectral mismatch. The photostability of devices was measured under AM 1.5G one sun illumination in N_2_ or ambient air condition. EQE spectra were measured under the short‐circuit condition using the Newport QUANTX‐300. The monochromated light was generated by a 5 Hz chopper frequency, and a silicon photodiode was used as a reference. Nonmodulated impedance spectroscopy was performed using the impedance analyzing function of an organic semiconductor parameter test system (McScience T4000) at various forward biases. A 30 mV voltage perturbation was applied over a constant forward applied bias between 0 and 1.0 V in the frequency range between 0.1 Hz and 1.0 MHz.

*General Characterizations*: UV–vis spectra were obtained using the Scinco S‐3100 UV–vis spectrophotometer. PL measurements were done with the Darsapro‐5000 (PSI Co. Ltd) spectrometer and a 325 nm monochromatic light as an excitation source and a charge coupled device as detector arrays. SCLC was measured using electron‐only device (ITO/ZnO/LiF/Al). Field‐emission SEM (JEOL JSM‐7610F) was used for imaging the surface of ZnO films. The AFM images (scan area: 5 µm × 5 µm) were acquired using a Seiko E‐Sweep atomic force microscope in tapping mode. UPS was performed using an AXIS‐NOVA (Kratos) system with HeI and a base pressure of 5 × 10^−8^ Torr. The samples were spin coated on Au substrates. XPS measurements were done with a MultiLab 2000 (THERMO VG SCIENCE) system equipped with Al *K_α_* radiation at *h*_ν_ = 1000 eV. The base pressure was 1 × 10^−9^ Pa. The binding energies were calibrated using the C 1*s* peak at 285.0 eV. The resulting spectra were analyzed using the CasaXPS software package with the Shirley method as the base line. The TEM specimens were prepared by dropping each ZnO sol–gel solution onto a copper grid. GIXRD measurements were conducted at 9A beam line of the Pohang Accelerator Laboratory in South Korea. The X‐rays were monochromated at wavelength λ = 0.62 Å using a double crystal monochromator, and the incidence angle of X‐ray beam was set to be 0.10° with sample‐to‐detector distance was 0.225 m.

## Conflict of Interest

The authors declare no conflict of interest.

## Supporting information

Supplementary
