# Supplementary material for: 11% Organic Photovoltaic Devices Based on PTB7‐Th: PC71BM Photoactive Layers and Irradiation‐Assisted ZnO Electron Transport Layers
Source: Adv Sci (Weinh). 2018 May 21;5(7):1700858. doi: 10.1002/advs.201700858 (PMC6051392; doi:10.1002/advs.201700858)
Supplement: Supplementary file 1 — Supplementary [file ADVS-5-1700858-s001.pdf]

Copyright WILEY-VCH Verlag GmbH & Co. KGaA, 69469 Weinheim, Germany, 2018.

Supporting Information

for *Adv. Sci.,* DOI: 10.1002/advs.201700858

11% Organic Photovoltaic Devices Based on PTB7-Th: $PC_{71}BM$ Photoactive Layers and Irradiation-Assisted ZnO Electron Transport Layers

Havid Aqoma, Sujung Park, Hye-Yun Park, Wisnu Tantyo Hadmojo, Seung-Hwan Oh, Sungho Nho, Do Hui Kim, Jeonghoon Seo, Sungmin Park, Du Yeol Ryu, Shinuk Cho, and Sung-Yeon Jang**

Copyright WILEY-VCH Verlag GmbH & Co. KGaA, 69469 Weinheim, Germany, 2016.

Supporting Information

11% Organic Photovoltaic Devices based on PTB7-Th:$PC_{70}BM$ Photoactive Layers and Irradiation-Assisted ZnO Electron Transport Layers

Havid Aqoma, Sujung Park, Hye-Yun Park, Wisnu Tantyo Hadmojo, Seung-Hwan Oh, Sungho Nho, Do Hui Kim, Jeonghoon Seo, Sungmin Park, Du Yeol Ryu, Shinuk Cho, Sung-Yeon Jang**

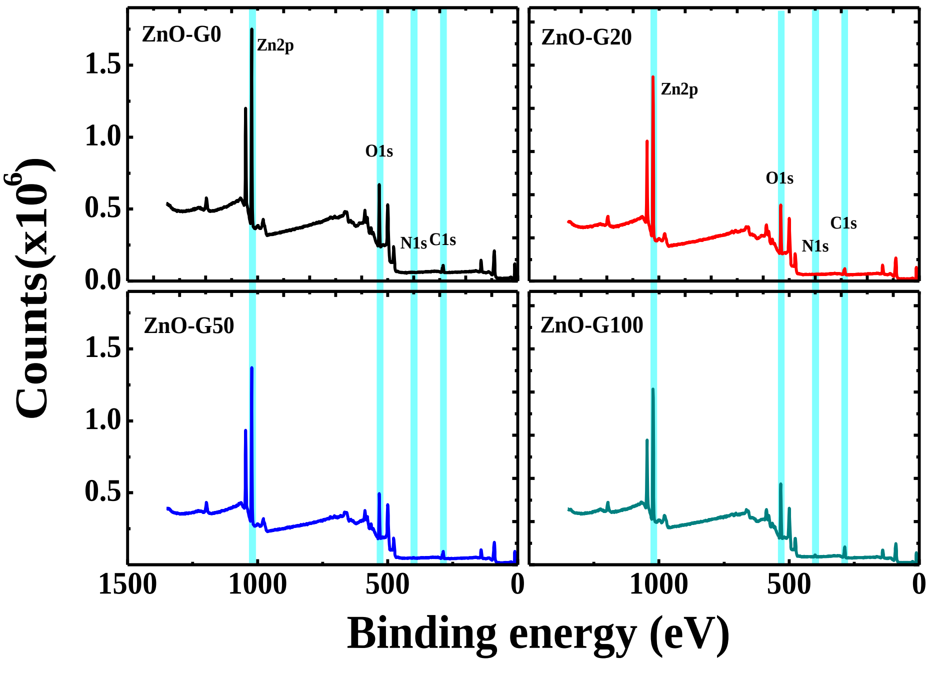

Figure S1. Wide-range XPS spectra for various ZnO films

Table S1. O 1*s* XPS analysis result of the ZnO films

	ZnO-G0 (%)	ZnO-G20 (%)	ZnO-G50 (%)	ZnO-G100 (%)
**O-non lattice-1**	34.83	35.27	27.75	33.06
**O-non lattice-2**	44.49	42.85	46.48	40.53
**O-lattice**	20.67	21.88	25.77	26.41
**Defect ratio**	3.84	3.57	2.88	2.79

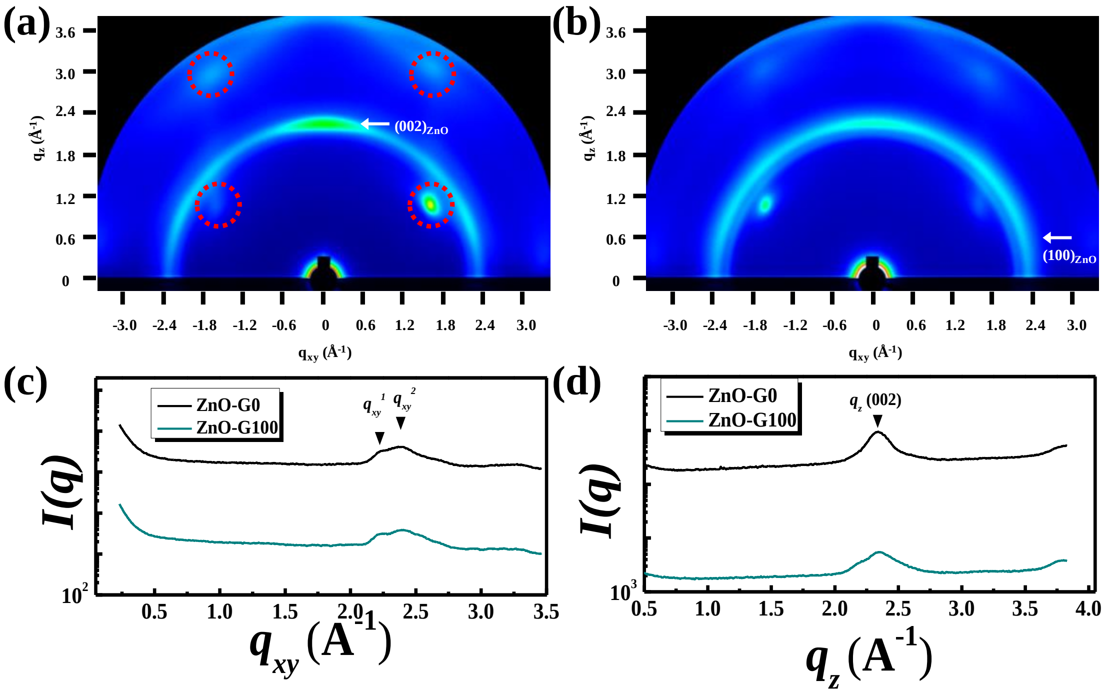

Figure S2. (a-b) Two-dimensional (2D) GIXRD images of ZnO films coated on SiO_2 substrate. (c) In-plane line-cuts and (d) out-plane line-cuts of 2D GIXRD images.

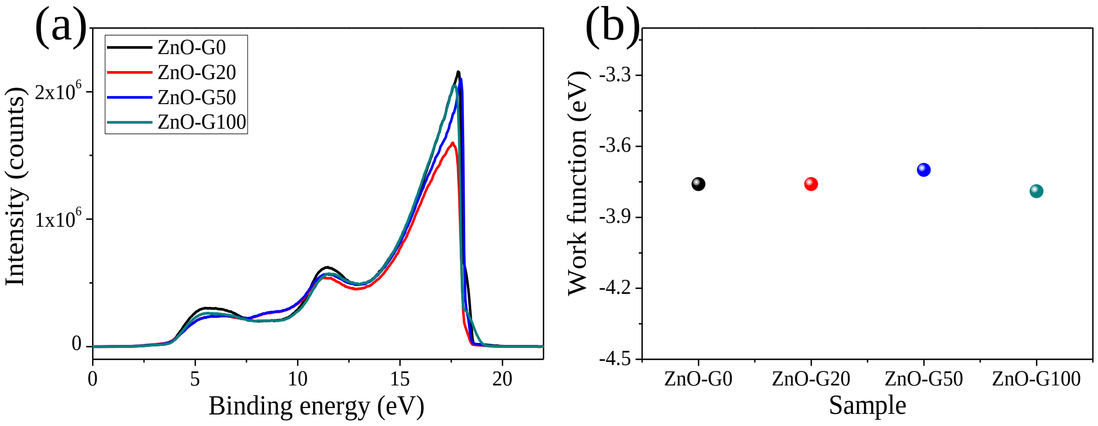

Figure S3. UPS analysis results of various ZnO films.

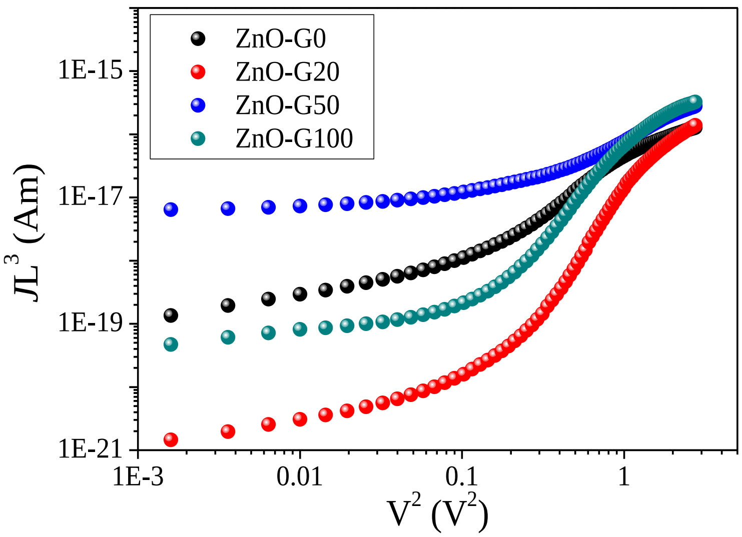

Figure S4. SCLC analysis results of electron-only devices using various ZnO.

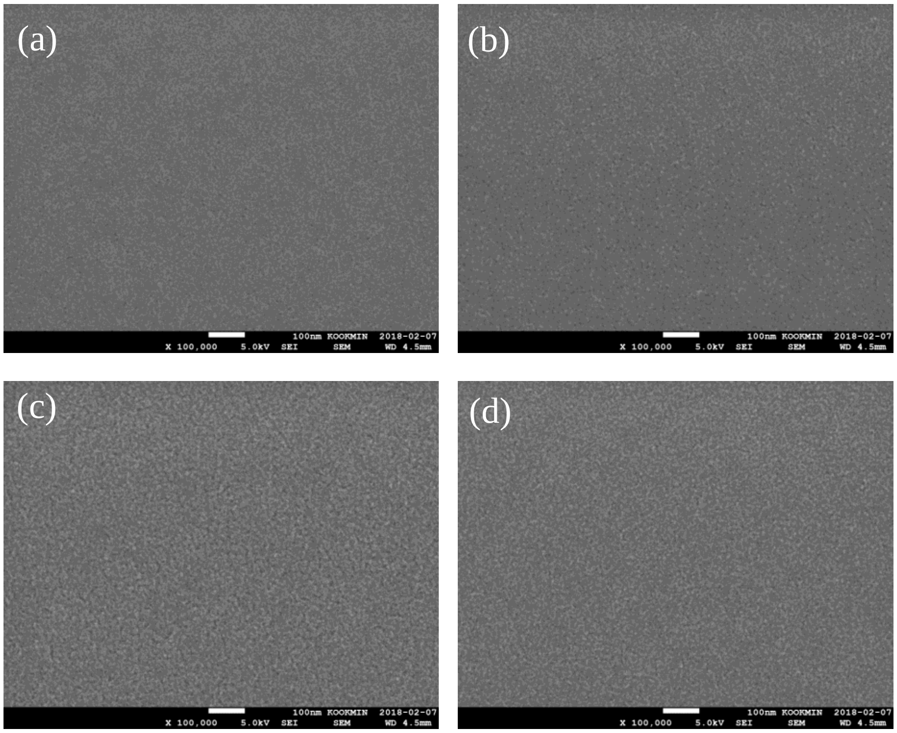

Figure S5. SEM images of (a) ZnO-G0, (b) ZnO-G20, (c) ZnO-G50, and (d) ZnO-G100 film surface.

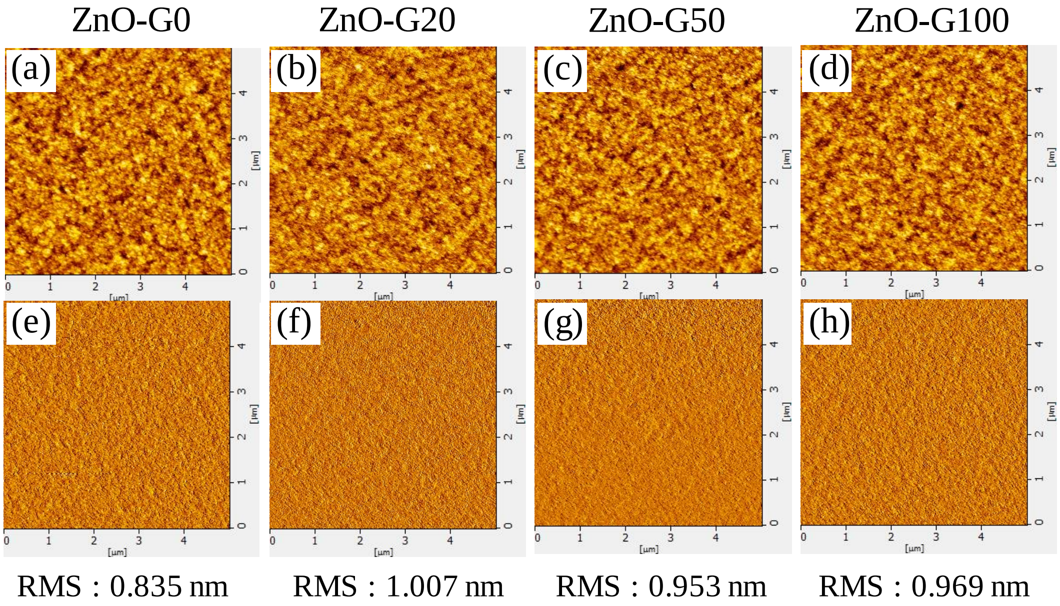

Figure S6. (a-d) Topographic images and (e-h) phase images of various ZnO films from AFM measurement.

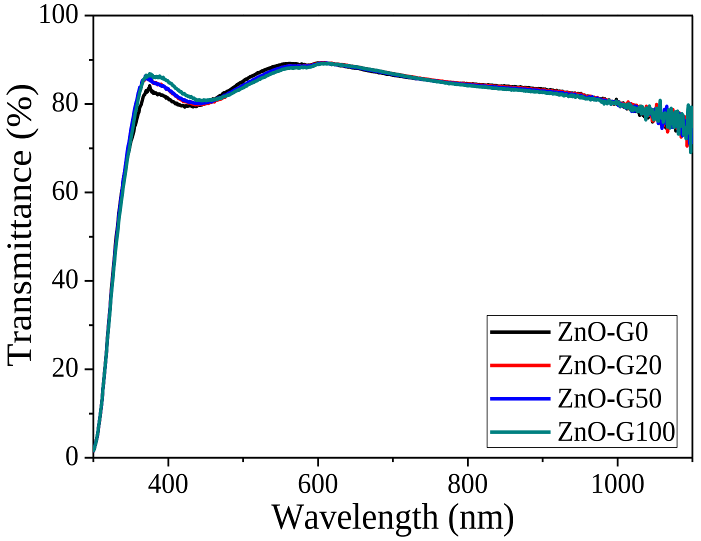

Figure S7. Transmittance spectra from UV-visible measurement of various ZnO films.

Table S2. Comparison of reported PCEs of OPV devices using PTB7-Th:$PC_{71}BM$ active layers and modified ZnO ETLs.

ETL	Initial PCE (%)	PCE modification (%)	Year	Reference
ZnO/PEIE	7.67	8.04	2016	[1]
ZnO-C60	7.64	9.35	2013	[2]
GZnO	8.69	9.83	2016	[3]
ZnO patterned	8.46	10.10	2014	[4]
ZnO/IL	8.94	10.15	2015	[5]
ZnO-open-PCBM	9.51	10.30	2016	[6]
ZnO-PbI-H	8.33	10.31	2015	[7]
InZnO-BisC60	8.25	10.31	2014	[8]
AlZnO/C-dots	9.05	10.32	2016	[9]
ZnO/PDEPB	8.70	10.40	2016	[10]
AZnO	8.93	10.42	2016	[11]
ZnO-2-naphthol/Ether	8.58	10.50	2016	[12]
ZnO-PbI	8.45	10.59	2015	[13]
ZnO-PEOz	8.81	10.70	2015	[14]
ZnO-Gamma ray	8.41	11.09	2018	Our work

Supplementary information references

[1] X. Jia, N. Wu, J. Wei, L. Zhang, Q. Luo, Z. Bao, Y.-Q. Li, Y. Yang, X. Liu, C.-Q. Ma, *Org. Electron.* **2016**, *38*, 150.

[2] S.-H. Liao, H.-J. Jhuo, Y.-S. Cheng, S.-A. Chen, *Adv. Mater.* **2013**, *25*, 4766.

[3] J. Wang, C. Yan, X. Zhang, X. Zhao, Y. Fu, B. Zhang, Z. Xie, *J. Mater. Chem. C* **2016**, *4*, 10820.

[4] J.-D. Chen, C. Cui, Y.-Q. Li, L. Zhou, Q.-D. Ou, C. Li, Y. Li, J.-X. Tang, *Adv. Mater.* **2015**, *27*, 1035.

[5] W. Yu, L. Huang, D. Yang, P. Fu, L. Zhou, J. Zhang, C. Li, *J. Mater. Chem. A* **2015**, *3*, 10660.

[6] C.-Z. Li, J. Huang, H. Ju, Y. Zang, J. Zhang, J. Zhu, H. Chen, A. K. Y. Jen, *Adv. Mater.* **2016**, *28*, 7269.

[7] L. Nian, W. Zhang, S. Wu, L. Qin, L. Liu, Z. Xie, H. Wu, Y. Ma, *ACS Appl. Mater. Interfaces* **2015**, *7*, 25821.

[8] S.-H. Liao, H.-J. Jhuo, P.-N. Yeh, Y.-S. Cheng, Y.-L. Li, Y.-H. Lee, S. Sharma, S.-A. Chen, *Sci. Rep.* **2014**, *4*, 6813.

[9] X. Lin, Y. Yang, L. Nian, H. Su, J. Ou, Z. Yuan, F. Xie, W. Hong, D. Yu, M. Zhang, Y. Ma, X. Chen, *Nano Energy* **2016**, *26*, 216.

[10] S. Nam, J. Seo, H. Han, H. Kim, S. G. Hahm, M. Ree, Y.-S. Gal, T. D. Anthopoulos, D. D. C. Bradley, Y. Kim, *Adv. Mater. Interfaces* **2016**, *3*, 1600415.

[11] X. Liu, X. Li, Y. Li, C. Song, L. Zhu, W. Zhang, H.-Q. Wang, J. Fang, *Adv. Mater.* **2016**, *28*, 7405.

[12] P. Fu, X. Guo, B. Zhang, T. Chen, W. Qin, Y. Ye, J. Hou, J. Zhang, C. Li, *J. Mater. Chem. A* **2016**, *4*, 16824.

[13] L. Nian, W. Zhang, N. Zhu, L. Liu, Z. Xie, H. Wu, F. Würthner, Y. Ma, *J. Am. Chem. Soc.* **2015**, *137*, 6995.

[14] S. Nam, J. Seo, S. Woo, W. H. Kim, H. Kim, D. D. C. Bradley, Y. Kim, *Nat. Commun.* **2015**, *6*, 8929.
